# Physiotherapeutic Approach in Oral Submucous Fibrosis: A Systematic Review

**DOI:** 10.7759/cureus.48155

**Published:** 2023-11-02

**Authors:** Neha M Chitlange, Pratik Phansopkar

**Affiliations:** 1 Department of Musculoskeletal Physiotherapy, Ravi Nair Physiotherapy College, Datta Meghe Institute of Higher Education and Research (Deemed to be University), Wardha, IND

**Keywords:** mouth opening exercise, osmf, ultrasound therapy, oral physiotherapy treatment, oral submucous fibrosis

## Abstract

One of the most poorly recognized and inadequately managed diseases, oral submucous fibrosis progresses over time. Betel nut eating is the foremost cause of oral submucous fibrosis. One such condition is oral submucous fibrosis, which is characterized by severe trismus, disability, and a higher risk of cancer. The mouth opening gradually decreases, which is related to difficulty eating, altered gustatory sensation, and mouth dryness, leading to decreased oral intake. The main and beginning signs include decreased mouth opening, pain, difficulty eating, dry mouth, and blanching of the buccal mucosa. This is treated first with medication, then with exercises for the mouth that a physiotherapist has recommended. However, the function of a physical therapist is not clearly defined. Physiotherapy may be used with other therapies to treat oral submucous fibrosis. Mouth-opening exercises, ultrasound, and other therapeutic interventions are available. This article tries to describe the kind of physical therapy that can be recommended for treating oral submucous fibrosis. It is crucial to understand pain management, physiotherapy management for grade III and IV oral submucous fibrosis various additional exercises, modalities and their ideal dose, and strategy for the long-term effect of the treatments to conduct further research.

## Introduction and background

Described by Pindborg et al. as “Epithelial degeneration causes discomfort in the oral mucosa, which results in trismus and the inability to eat. It always coexists with a juxta epithelial inflammatory reaction followed by fibroelastic change of the lamina propria” [1]. The consistent chewing of areca nuts is a significant contributor to the development of oral submucous fibrosis [2]. Insufficient health, chilly intake, genetic susceptibility, autoimmune disease, and collagen problems are some of the additional risk factors [3]. Although it is more common in the Indian subcontinent, oral submucous fibrosis is a potentially fatal disease now widespread worldwide [4]. Many individuals are sensitive to spicy foods and lip, tongue, and palate stiffness, resulting in varying degrees of open mouth and tongue mobility limitation. The condition's hallmark is submucosal fibrosis, primarily affecting the mouth, pharynx, and upper one-third of the esophagus [5]. With an estimated incidence of up to 0.4% in rural areas, India has the highest prevalence of this precursory condition, followed by Bangladesh, Sri Lanka, Pakistan, Taiwan, and China. In India, Bihar, Maharashtra, Gujarat, and Madhya Pradesh all witness oral submucous fibrosis [6]. In India, the incidence of oral submucous fibrosis varies between 0.2% and 0.5%, with the southern region having the highest plurality [7]. Ages 11 to 60, men's oral submucous fibrosis prevalence figures in India range from 0.2% to 2.3% and 1.2% to 4.6% for women [8]. Oral submucous fibrosis is primarily treated with dental treatments, but physical therapy is also essential. Physical therapy has a significant impact because opening the mouth has a restricted range of motion. Additionally, it might be challenging to complete these exercises solely if the fibrosis is severe [9]. Physical therapy adds another dimension to treatment in addition to surgery and medication [10]. Beneficial results have Pathophysiology of oral submucous fibrosis been achieved when medication and physical therapy are combined. The treatment of oral submucous fibrosis has been presented to be significantly impacted by exercise [11].

Several theories have been put forth, indicating multifactorial origins, but the cause of oral submucous fibrosis is still unknown. One of the most important causes has been linked to consuming areca nut and betel quid [12]. Reactive oxygen species, matrix metalloproteinases, copper-lysis oxidase enzymes, and autoimmune and genetic variation are just a few of the processes and molecules that contribute to oral submucous fibrosis's etiology [12]. The arecoline component of betel nuts plays a significant role in the disease progression of oral submucous fibrosis by producing free radicals and reactive oxygen species and being essential in regulating inflammatory mediators and growth factors [13]. Based on circulatory immune complexes, antibody contents, the identification of autoantibodies in the serum, variations in cellular and humoral responses, and the involvement of the DR locus in the genetic predisposition, autoimmunity as an etiological cause of oral submucous fibrosis can be taken into consideration [14]. Clinical manifestation of oral submucous fibrosis Over a two- to five-year duration, the beginning is gradual [15]. The oral mucosa blanch is the first clinical mark of oral submucous fibrosis, and fibrous bands start forming in the affected areas. The bars typically affect the tongue, lips, posterior pharynx, palate, buccal tissue, and palate [16]. It burns when eating hot, spicy food, and a gradual decline in mouth opening are the most typical presenting symptoms, which are also linked to difficulty swallowing, altered taste sensation, dry mouth, and nasal voice [17]. In the early stages of the oral mucosa, ulcers and blisters are visible. Small blisters on the cheek and palate indicate worsening phases [16]. The oral mucosa becomes blanched, slightly translucent, and shows white fiber bands as the condition worsens [15].

Grades of oral submucous fibrosis

Oral submucous fibrosis is classified under various categories such as functional grading, clinical grading, and histopathological staging. There are four grades of oral submucous fibrosis as per the severity of the disease. The different grading shows the severity and involvement of buccal mucosa of the oral cavity in oral submucous fibrosis. All the features of oral submucous fibrosis are described in Table 1 [18]. Grade 1 is the mild form of oral submucous fibrosis and grade 4 is the very severe form of oral submucous fibrosis as it may show malignant changes.

**Table 1:** Based on functional grading, clinical grading, and histopathological staging

Grading	Functional grading (mouth opening)	Clinical grading	Histopathological staging
Grade 1	Up to 35mm	Less than 1/3 of the buccal cavity is affected, and symptoms include stomatitis, mild blanching, burning, and recurrent ulcers. Xerostomia.	Fine edematous collagen, clogged blood vessels, a proliferation of neutrophils and lymphocytes, and myxomatous changes in the subepithelial connective tissue are all signs of an advanced stage of inflammation.
Grade 2	22-35mm, cheek elasticity decreased by 33%	1/3 to 2/3 of the buccal cavity is affected, and the soft palate and premolar region is involved, blanching, a marble-like look, and palpable fibrotic bands.	White Blood Cell's like Lymphocytes and eosinophils, enlarged and clogged blood vessels, less fibroblastic activity, and juxta-epithelial collagen hyalinization. Reduced inflammatory cells in the subepithelial layer and granular changes in the muscle layer
Grade 3	15-25mm, cheek elasticity decreased by 66%	More than two-third of the oral cavity depapillated tongue and limited tongue movement shrunken bud like uvula, the floor of the mouth involvement, broad, thick fibrous palpable bands at cheeks and lips, severe blanching, ang lymphadenopathy	Stages of fibrosis include total collagen hyalinization without fibroblasts or oedema, the obliteration of blood vessels, plasma cells, and lymphocytes, and extensive fibrosis with hyalinization from subepithelial to superficial muscle layers along with atrophic, degenerative changes.
Grade 4	<15mm or nil	Alterations such as leukoplakia, erythroplakia that is ulcerating, and a suspicious malignant lesion	Malignant development in stages: erythroplakia develops into carcinoma of squamous cells.

Assessment

A thorough history of the patient's primary complaint must be kept along with the habit history. On a scale of 0 to 10, where 0 denoted no burning sensation and 10 the worst burning sensation imaginable, a visual analog scale was used to assess the extent of the burning sensation. The mesio-incisal angles of the bottom central incisor and the upper central incisor were measured using a vernier caliper. The mouth opening, also known as the inter-incisal distance, was measured and noted in millimeters.

Diagnostic investigations

 A clinical diagnosis only requires history, clinical evaluation, and signs and symptoms. A helpful clinical test is pain in the areas where submucosal fibrotic bands are evolving when palpated [15]. However, a histopathological examination is necessary to determine the stage of development [1].

Physiotherapy treatment

Numerous safe treatment options may be able to produce the desired effects. Ultrasound has been used successfully and frequently in physical therapy [19]. Ultrasound therapy increases cell membrane permeability by modifying the sodium and potassium ion gradients. The improved gas exchange and accelerated healing caused by this raised permeability [20]. Vasodilatation, waste removal, and inflammation are all reduced by ultrasound [21]. Increases lymph flow and metabolism. Ultrasound therapy reduces pain, hastens the healing process, and improves collagen fiber extensibility [22].

Kneading is a beneficial massage therapy technique that helps mobilize scar tissues and increase the flexibility of fibrous tissues [23]. In physical therapy, the gentle manipulation of soft tissues is frequently used to increase their flexibility. Forced passive movements and manipulations are used to mobilize the less mobile temporomandibular joint to increase mouth opening [21].

Patients with oral submucous fibrosis can perform various exercises to improve their flexibility and protrusion of their tongue [9]: (1) Tongue blade exercise, (2) tongue-in-cheek push, (3) side tongue stretch, (4) cheek puff, (5) pucker, (6) up and down tongue stretch, and (7) teeth sweep. Performing oral physical therapy exercises like the ice cream stick exercise five times daily is recommended for forceful mouth opening [23].

## Review

Methodology

**Figure 1:**
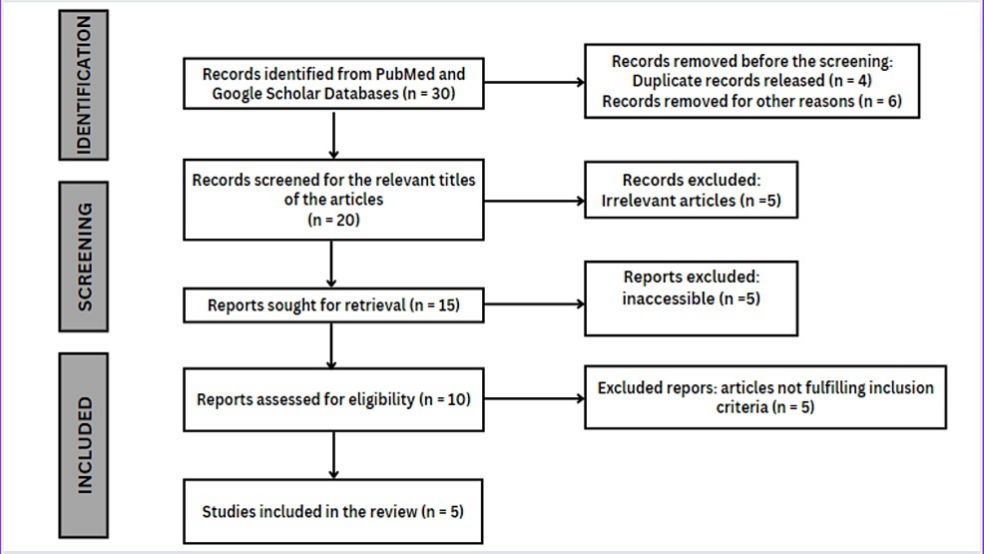
Preferred reporting items for systematic reviews and meta-analyses flowchart

Papers from 2009 to 2022 were discovered in databases like Web of Science, Scopus, Google Scholar, and Pubmed. Studies that are experimental trials were included. These research papers were chosen based on the criteria: experimental studies, physiotherapy, and rehabilitation should focus on mouth opening; articles should have been published between 2009 and 2022; and papers should have been published in English. The study did not include case series, cohorts, reviews, and non-experimental studies. Based on the criteria for inclusion, five articles that specifically emphasized mouth opening in cases of oral submucous fibrosis are mentioned in the study after the preliminary screening of 10 articles. Preferred reporting items for systematic reviews and meta-analyses flowcharts are shown in Figure 1. A summary of the articles like Thakur et al., Vijayakumar et al., Arora et al., Asha et al., and Cox et al. is reviewed for physiotherapy treatment for oral submucous fibrosis is shown in Table 2.

**Table 2:** Summary table of the articles included in review

Author and year of publication	Study type (sample size)	Intervention	Result	Conclusion
Nidhi Thakur et al.2011 [4]	randomized controlled trial (64 patients of Oral Submucous Fibrosis)	Group l- micronutrients and physical therapy exercises. Group II- physical therapy exercises. Group III- micronutrient supplements. And evaluated at 1, 3 & 6 weeks.	It used the Kruskal-Wallis test for statistical analysis. The mouth opening measurements for the group I individuals significantly increased over six weeks compared to the initial mouth opening. The slightest betterment was seen in Group II values. .	As the primary form of treatment, micronutrients and physical therapy should be used.
Vijayakumar M et al.2013 [21]	Prospective clinical trial. (15 individuals of Oral Submucous Fibrosis)	With (massage therapy) finger and thumb kneading, ultrasound for succession for two weeks.	The average mouth opening increased by 6.26mm. After the tenth day, there was a statistically significant difference in the mean mouth opening, according to the results of the Dunnett test for multiple comparisons.	They can open their mouth wider and experience less burning pain thanks to the successful role of physiotherapy intervention in managing Oral Submucous Fibrosis complications.
.Pooja K. Arora et al.2010 [10]	Randomized controlled trial (30 patients)	For seven days, 0.7 watts/cm2 of ultrasound therapy was administered every minute, followed by jaw-opening exercises. .	Before treatment, the average highest opening of the mouth was 24.06mm; after treatment, it was 27.8mm.	Exercises using ultrasound to open the mouth can be a different type of treatment for trismus.
Asha V et al. 2017 [24]	Randomized controlled trial (48 patients)	Group 1 intralesional injections of dexamethasone and hyaluronidase. Group 2: oral physical therapy Group 3 treatments include oral physical therapy and intralesional injections.	Group 3 had the highest mean differences in oral rehabilitation	The mean mouth opening, tongue protrusion, and cheek flexibility improved with simultaneous rehabilitation and intralesional injections.
Stephen Cox et al. 2009 [25]	Randomized controlled trial (54 patients)	Group 1: rehabilitation using tongue spatulas placed alternately between teeth. Group 2: local hyaluronidase and steroid injection. Group 3 receives no active therapy.	The oral opening was improved by physical therapy but not oral pain, and both untreated and injectable patient groups showed no discernible enhancement.	The mouth opening can be increased with physical therapy.

Discussion

Oral submucous fibrosis is one of the most popular premalignant diseases that may impact the buccal cavity in people who consume little nuts. In India, the prevalence is between 0.2% and 0.5%, with a higher majority in the southern regions. Most affected people are between 20 and 40. However, cases in patients as young as two and as old as 89 have been reported [26]. Oral submucous fibrosis has been treated using a variety of methods, including both medical and surgical interventions. The most crucial therapy should be habit change because areca nut chewing has been identified as one of the key contributors to oral submucous fibrosis pathogenesis. Individuals should therefore be advised to break the habit [10]. Patients with severe trismus should consider surgery if other treatments have not successfully treated the condition. However, the outcomes are unsatisfactory because the healing of the wound has contracted, which has caused more fibrosis [27].

Currently, mouth-opening/exercising devices are used in oral physiotherapy as an adjunctive modality in treating oral submucous fibrosis [10]. It is rarely used as the primary method of increasing mouth opening. It has the advantages of being a safe approach, getting less damaging for the individual, and restricting monetary necessity [10]. Mouth exercises are beneficial as a procedure that patients will comply with because they can be done anywhere throughout the day. Researchers have found that using various physiotherapeutic modalities helps oral submucous fibrosis patients with their restricted mouth opening [4].

Usually, physical therapy is added after surgery to help patients open their mouths wider using tongue spatulas and jaw-opening instruments. The effectiveness of this oral rehabilitation in treating individuals who had oral submucous fibrosis was evaluated, and it was discovered that patients who performed mouth exercises had an average rise in mouth opening, tongue protrusion, and cheek flexibility [10].

For mild to moderate cases, several studies have been conducted in the past to evaluate the efficacy of physiotherapy [28]. Physical therapy can be an adjunct to micronutrients in a survey on oral submucous fibrosis patients [4]. Another oral submucous fibrosis study used physiotherapy and ultrasound therapy to improve mouth opening significantly [21]. In cases of oral submucous fibrosis undergoing surgery, oral physiotherapy substantially lowers the risk of scar contracture and relapse [25].

In the past, effective medical treatments such as corticosteroids, placental extracts, hyaluronidase, pentoxifylline, etc. have been used [29]. Patients with severe trismus should consider surgery if other treatments have not been successful in treating the condition. However, the outcomes are unsatisfactory because the healing of the wound is contracting, which causes more fibrosis [27]. Studies utilizing various physiotherapeutic modalities have demonstrated an improvement in patients with oral submucous fibrosis's restricted mouth opening [25]. Physical therapy has been applied using tools that open the mouth and jaw violently.

Study limitation

Fewer studies have been done on physiotherapy in oral submucous fibrosis for grade III and IV mouth opening, so more research is needed. It is challenging to manage the pain; more research is required to determine the affordability of physiotherapeutic treatment for oral submucous fibrosis.

## Conclusions

Physical therapy may be used with other treatments to treat oral submucous fibrosis. Patients prefer it because it is independent, economical, safe, free of side effects, and less damaging. As a result, it should be used as the primary modality in rehabilitating oral submucous fibrosis. It is possible to more successfully supervise the clinical symptoms and signs of oral submucous fibrosis individuals by integrating these treatments with patient determination. It can also be used as a first line of treatment for patients who refuse intralesional steroids. Individuals prefer physical therapy more because it is non-traumatic and requires fewer resources.
